# The Role of Tyrosine and C-Reactive Protein in COPD Exacerbations

**DOI:** 10.3390/jcm14248933

**Published:** 2025-12-17

**Authors:** Ping-Chi Liu, Chao-Hung Wang, Wan-Chi Lin, Wei-Ke Kuo

**Affiliations:** 1 Division of Pulmonary, Critical Care, and Sleep Medicine, Chang Gung Memorial Hospital, Keelung 204, Taiwan; ewind14@hotmail.com; 2 College of Medicine, Chang Gung University, Taoyuan 333, Taiwan; bearty54@gmail.com; 3 Heart Failure Research Center, Division of Cardiology, Department of Internal Medicine, Chang Gung Memorial Hospital, Keelung 204, Taiwan; 4 Department of Anesthesiology, Sijhih Cathay General Hospital, New Taipei 221, Taiwan; 5 Division of Respiratory Therapy and Chest Medicine, Department of Internal Medicine, Sijhih Cathay General Hospital, New Taipei 221, Taiwan; 6 Department of Bioscience and Biotechnology, National Taiwan Ocean University, Keelung 202, Taiwan

**Keywords:** COPD, C-reactive protein, exacerbations, metabolomics, tyrosine

## Abstract

**Background/Objectives**: Chronic Obstructive Pulmonary Disease (COPD) exacerbations affect health and mortality, yet current risk assessments based on previous events have limitations and are less preventative. This study used metabolomics to assess if amino acid profiles are linked to higher COPD exacerbation risk, compared to an amino acid-based panel to standard risk stratification methods, and explored its clinical decision-making and prevention potential. **Methods**: This prospective cohort study measured plasma concentrations of 19 amino acids in 88 individuals with COPD using ultra-performance liquid chromatography. Participants were observed for 2.5 years to track occurrences of moderate and severe COPD exacerbations. **Results**: During follow-up, 44 participants (50%) had an exacerbation. Tyrosine and hsCRP were independently linked to exacerbations in multivariable analysis and formed the basis of the “COPDAE score.” This score independently predicted future exacerbations after adjusting for Global Initiative for Chronic Obstructive Lung Disease (GOLD) classifications. A COPDAE score above 1.73 correlated with lower event-free survival in several subgroups: GOLD group A (log-rank = 13.7, *p* < 0.001), groups A and B (log-rank = 5.0, *p* = 0.025), and groups A and C (log-rank = 15.2, *p* < 0.001). **Conclusions**: Tyrosine and hsCRP together form a biomarker panel to assess and stratify COPD exacerbation risk. This score can help identify stable patients at risk, even with mild symptoms or few prior events, enabling earlier, more personalized interventions.

## 1. Introduction

Chronic obstructive pulmonary disease (COPD), a major cause of chronic morbidity and mortality worldwide, is a great economic and social burden [1]. COPD exacerbations, defined as acute worsening of respiratory symptoms that results in additional therapy [2], are associated with gas trapping, increased mucous production, and airway inflammation which cause increased dyspnea, sputum volume and purulence, cough, and wheezing [3]. COPD exacerbations are important events in the management of COPD because they negatively impact health status, disease progression, and rates of rehospitalization [4,5]. The five-year mortality rate following COPD exacerbation-related hospitalizations is about 50% [6]. Identifying patients at risk of developing COPD exacerbation is important as it could assist in guiding preventive strategies.

Risk factors for COPD exacerbation include percentage of predicted forced expiratory volume in 1 s (FEV1), older age, productive cough, duration of COPD, systemic corticosteroid or antibiotics use for COPD in the prior year, theophylline use, and hospitalization for COPD in the prior year [7,8]. Biomarkers demonstrated to predict COPD exacerbation include C-reactive protein (CRP), blood eosinophil level, fibrinogen level, and soluble urokinase-type plasminogen activator receptor [9,10,11,12]. Although these biomarkers provide useful clinical information, no single one of them has gained wide acceptance. Currently, the number of exacerbations of patients who have had COPD in the past year remains the strongest predictor of their future exacerbations [13]. The Global Initiative for Chronic Obstructive Lung Disease (GOLD) guidelines suggest using a combination of symptoms and history of exacerbations and hospitalizations for exacerbation to assess the risk of exacerbation and guide medication treatment [14]. However, passive adjustment of medication solely on prior exacerbations may increase the risk of future episodes, because exacerbation is itself a strong risk factor of subsequent events in COPD. In other words, preventing exacerbations before they happen could improve clinical outcomes. Consequently, objective biomarkers for assessing the risks of COPD exacerbations would serve as a valuable tool to enable clinicians to adjust medication and prevent future events.

Metabolomics is a minimally invasive and sensitive approach to investigating the biochemical changes in body fluids, tissue and cells to represent an individual’s health status and help disease management [15]. It has been increasingly used in COPD-associated studies on pathogenesis, diagnosis, treatment, and prognosis [16,17]. Few studies have focused on using metabolomics-based factors to assess the risk of COPD exacerbations. Wassim et al. reported that lower concentrations of serum amino acids including tryptophan, leucine, isoleucine and valine were independently associated with 1-year incidence of respiratory exacerbations. However, only 56.8% and 58.0% of study populations were current smokers and had spirometry-confirmed COPD [18]. Previously, we utilized ultra-performance liquid chromatography (UPLC) to identify specific amino acid alterations associated with advanced COPD [19]. Although untargeted metabolomics offers a broader discovery scope, it poses challenges for clinical standardization [20]. In contrast, UPLC-based amino acid profiling provides a rapid, simple, and quantifiable approach ideal for routine risk assessment [21]. Furthermore, COPD involves key pathophysiological characteristics such as systemic inflammation and metabolic reprogramming processes that are sensitively reflected in amino acid turnover [22]. Therefore, this study employs this targeted, clinically feasible approach to determine if specific amino acid profiles can predict future exacerbations.

The aims of this study were (I) to investigate whether specific amino acids are associated with an increased risk of COPD exacerbations; (II) to assess the utility of an amino acid-based panel in evaluating the risk of COPD exacerbations compared to traditional assessment tools, such as GOLD groups or the BODE index [23]; and (III) to examine the potential for the amino acid-based panel to enhance existing assessment methods and assist clinical decision-making in identifying and managing the risk of COPD exacerbations.

## 2. Materials and Methods

### 2.1. Patients and Study Design

Between May 2017 and July 2019, patients aged 40 to 90 years who met the GOLD criteria for COPD were consecutively enrolled from outpatient settings. Exclusion criteria included the following: patients with a serum creatinine of >3 mg/dL; patients who were bed-ridden for more than three months and/or unable to stand alone; patients with disorders that might compromise their survival within the next six months; patients with active cancer. We used convenience sampling without formal sample size calculation to ensure logistical feasibility for the specialized metabolic analysis and long-term prospective follow-up. Informed consent was obtained from all patients. The study was designed and carried out with approval from the Ethics Review Board of Chang Gung Memorial Hospital (IRB/CGMH, No.201700159B0C102) and in accordance with the principles of the Declaration of Helsinki.

### 2.2. Clinical Variables Related to COPD

The following variables were recorded in study patients: age; sex; comorbidities; body mass index; FEV1 [24]; modified Medical Research Council questionnaire (mMRC) [25]; chronic obstructive lung disease assessment test (CAT) [26]; six-minute walking distance (6MWD) [27]; hand grip strength of the dominant hand [28]; oxygen saturation (SpO2); GOLD groups (patients were classified into GOLD groups A, B, C, and D based on the guidelines current during the study period (2017–2019). We maintain this four-group classification (A-D) for consistency, despite the later simplification of the GOLD guidelines (2024–2025) to three groups (A, B, E) [29,30]; BODE index (BODE stages 1, 2, 3 and 4 were defined by BODE 0–2, 3–4, 5–6, and 7–10 points, respectively); COPD medication (inhaler and oral corticosteroids).

### 2.3. Blood Sampling and Examination

To measure metabolites, blood samples of patients we collected in Ethylenediaminetetraacetic Acid (EDTA)-containing tubes in the early morning after overnight fasting. Plasma was analyzed by UPLC workflow. Measurement of other parameters was conducted in the central core laboratory, including high-sensitivity C-reactive protein (hsCRP), albumin, pre-albumin, and transferrin.

### 2.4. Follow-Up Program

Follow-up data were prospectively obtained monthly through personal communication with the patients’ physicians, regular patient visits to outpatient clinics and hospital records. Patients were followed for up to 2.5 years until the occurrence of death, study completion, or loss of follow-up. The primary outcome was the composite event of moderate or severe COPD exacerbations, defined according to GOLD guidelines for prognostic analysis. Moderate exacerbations required treatment with short-acting bronchodilators plus antibiotics and/or systemic steroids, while severe exacerbations required hospitalization or an emergency room visit [14]. To ensure accuracy, all exacerbation events were verified by cross-referencing hospital medical records and consulting with the patients’ primary care physicians.

### 2.5. UPLC-Based Measurement

The plasma samples (100 µL) were precipitated by 10% sulfosalicylic acid. After protein precipitation and centrifugation, we performed AQC derivatization in acetonitrile. Amino acids were analyzed on a ACQUITY UPLC system (Waters Corp., Milford, MA, USA) composed of a sample manager, a binary solvent manager, and a tunable UV detector. The system was controlled, and data was collected using Empower™ 2 Software (Waters Corporation, Milford, MA, USA). Separations were performed on a 2.1 × 100 mm ACQUITY BEH C18 column (Waters Corp.) at a flow rate of 0.70 mL/min.

The method validation results for all amino acids were as follows: The range of average intra-assay coefficients of variation (CV) was 4.3% to 4.6%, the range of total CV was 3.1% to 4.1%. The limit of detection range was 0.5 micromolar (μM) to 3.3 μM, and the linear range was 25 to 500 μM.

### 2.6. Statistical Analysis

We used SPSS 22.0 (SPSS Software, Chicago, IL, USA) to analyze data. Categorical variables are presented as numbers (percentages), while continuous variables are expressed as mean ± standard deviation or median (interquartile range). Data were compared using independent *t*-test where appropriate. To determine independent predictors of the first defined events (composite event of moderate and severe exacerbations), we used Cox proportional hazard models with forward stepwise analysis. Variables with a *p* value < 0.1 were selected for the multivariable analysis. We use Cox proportional hazards regression to calculate Hazard Ratios (HRs) and 95% Confidence Intervals (CIs). Specifically, we developed the “COPDAE score” using the β estimates derived from multivariable Cox regression model. The optimal cutoff value for the COPDAE score was determined using Youden’s index calculated from Receiver Operating Characteristic (ROC) curves. Time-dependent outcomes were compared using Kaplan–Meier survival analysis with a log-rank test. A *p* value of <0.05 was considered statistically significant.

## 3. Results

### 3.1. Baseline Characteristics

A total of 88 patients were enrolled in the study. Over a 2.5-year follow-up period, 44 patients (50.0%) had a composite event, including 8 moderate (9.0%) and 36 severe (40.9%) exacerbations. Baseline characteristics and laboratory data (Table 1) indicated that patients with exacerbation events had higher mMRC scores, lower oxygen saturation levels, and were more frequently classified in GOLD group D.

### 3.2. Identifying Amino Acids with Potential Prognostic Value

Table 2 presents the comparisons of all amino acids and laboratory parameters between patients with and without composite events. To identify prognostic factors, Cox univariate and multivariable analyses were performed. Results indicated that tyrosine and the logarithm of HsCRP (Log (HsCRP)) were associated with the prognosis of COPD exacerbations. These two variables were subsequently used to develop the “COPDAE score.” The score was constructed as a weighted linear combination of these variables, where the weights correspond to their respective regression coefficients (β) from the final multivariable model. The score was calculated as follows: (0.508 × Log (HsCRP)) + (0.024 × tyrosine).

**Figure 1:**
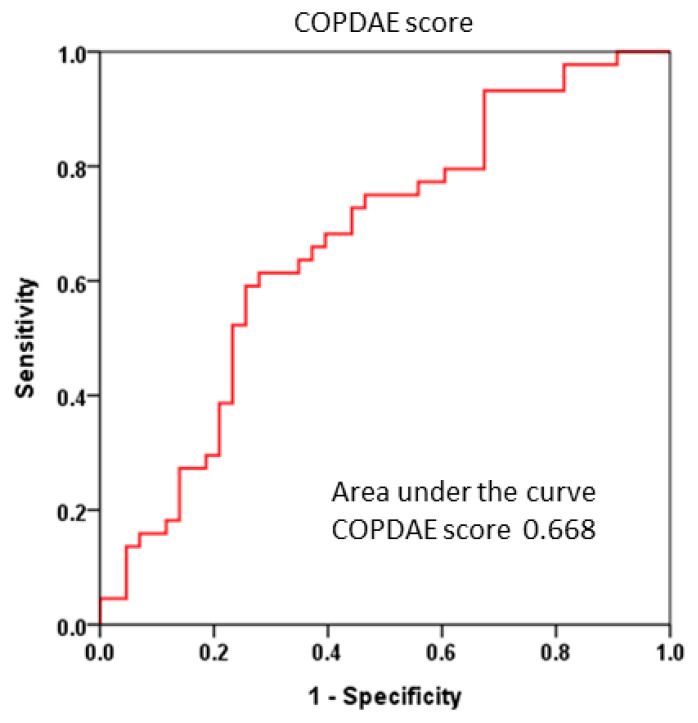
ROC curve analysis assessing the discriminative performance of the COPDAE score. The ROC analysis demonstrated an AUC of 0.668, indicating a moderate ability to differentiate between patients with and without composite events. ROC, Receiver Operating Characteristic. AUC, Area Under the Curve.

Table 3 presents the associations between several clinical parameters and composite events. Univariate analysis identified mMRC score, GOLD group classification, BODE stage, and SpO2 as variables significantly related to the occurrence of COPD exacerbations. The COPDAE score was then adjusted for these significant clinical parameters in multivariable analysis. Importantly, the COPDAE score remained independently associated with events, even after adjustment for GOLD group classification (Table 3). The ROC curve analysis demonstrated the score’s discriminative ability between patients with and without composite events, yielding an Area Under the Curve (AUC) of 0.668 (Figure 1). Based on Youden’s index, the optimal cutoff value for the COPDAE score was determined to be 1.73. A COPDAE score of >1.73 was significantly associated with a higher risk of composite events (HR: 3.33, 95% CI = 1.78–6.19, *p* < 0.01).

### 3.3. Prognostic Value of COPDAE Score and Existing Parameters for COPD AE

**Figure 2:**
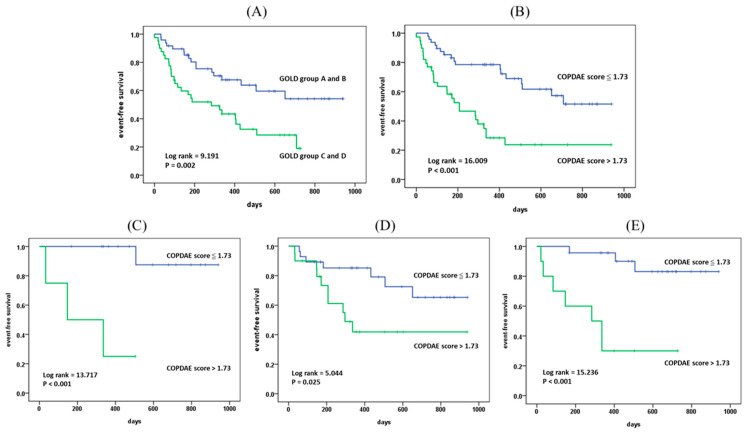
Kaplan–Meier survival curves illustrating risk stratification for composite events. (**A**) Event-free survival according to GOLD classification, with high-risk groups (**C**,**D**) showing significantly reduced event-free survival compared to low-risk groups. (**B**) Event-free survival curves based on COPDAE score using a cutoff of 1.73. Patients with a COPDAE score > 1.73 had markedly reduced event-free survival versus those with scores ≦ 1.73 (**C**) Event-free survival analysis within the GOLD A subgroup. The COPDAE score maintained significant prognostic value, as individuals with scores > 1.73 showed substantially decreased event-free survival. (**D**) Among GOLD A and B groups (fewer prior exacerbations), the COPDAE score effectively stratified risk for even-free survival. (**E**) In GOLD A and C groups (less severe symptoms), the COPDAE score also efficiently stratified risk for event-free survival. GOLD, Global Initiative for Chronic Obstructive Lung Disease.

The Kaplan–Meier survival curves (Figure 2) demonstrate the effectiveness of both GOLD classification and the COPDAE score in stratifying the risk of composite events. Patients classified into the high-risk GOLD groups C and D showed significantly lower event-free survival compared to the low-risk groups A and B (log rank = 9.19, *p* = 0.002; Figure 2A). Similarly, patients with a COPDAE score > 1.73 had significantly lower event-free survival compared to those with scores ≤ 1.73 (log rank = 16.1, *p* < 0.001; Figure 2B). The COPDAE score also maintained its significant prognostic value within clinically relevant subgroups. In GOLD A groups (representing patients with less severe symptoms and fewer past exacerbations), individuals with a COPDAE score > 1.73 exhibited a substantially lower event-free survival (log rank = 13.7, *p* < 0.001, Figure 2C), confirming the score’s ability to identify high-risk individuals among low-risk individuals. The COPD score also effectively stratified patients within the GOLD A and B groups (representing those with fewer past exacerbations) (log rank = 5.0, *p* = 0.025, Figure 2D), and the GOLD A and C groups (representing patients with less severe symptoms) (log rank =15.2, *p* < 0.001, Figure 2E). In all these subgroups, COPDAE score > 1.73 was associated with significantly lower event-free survival compared to those with ≤1.73.

## 4. Discussion

In this study, we found that elevated tyrosine and HsCRP levels were independently associated with a higher risk of future COPD exacerbations. Based on these findings, we developed the “COPDAE score” to assist clinicians in predicting and stratifying this risk. Furthermore, our findings suggest that the COPDAE score offers significant prognostic value even in COPD patients considered low-risk, such as those with fewer symptoms and a limited exacerbation history (GOLD group A). Although our study used the four-group GOLD classification (A, B, C, D) applicable during data collection (2017–2019), the COPDAE score remains a valuable prognostic tool under the current 2024 system (Groups A, B, and E). Since the current Group E encompasses the former high-risk Groups C and D, and the definition of the low-risk Group A remains unchanged, our finding that the score identifies hidden risk within Group A is directly applicable to modern practice.

COPD is primarily characterized by abnormal inflammatory response and airflow limitation. Exacerbations represent periods of increased inflammatory burden, which rapidly worsen respiratory symptoms [31]. The stability of airway inflammation is often disrupted by various triggers, including bacterial and virus infections, air pollution, temperature changes, gastroesophageal reflux, bronchiectasis and obstructive sleep apnea, all leading to acute COPD exacerbations [31]. This heightened inflammatory state, marked by an increased number of inflammatory cells and cytokines, stimulates the production of nitric oxide (NO) by airway epithelium, macrophages and vascular smooth muscle [32,33]. Accordingly, the amount of NO in exhaled air has been found to be substantially greater during acute exacerbations and in patients with more severe COPD [34].

Oxidative stress is a central characteristic of COPD, arising from an imbalance between antioxidant defenses and the production of reactive oxygen and nitrogen species (ROS/RNS). During COPD exacerbations, a distinct increase in both locally and systemically elevated oxidative stress is consistently observed [35]. Environmental stimuli, such as respiratory pathogens, air pollution, allergens and cigarette smoke, directly or indirectly increase cellular levels of ROS. Specifically, the rapid reaction of superoxide anions with nitric oxide (NO) leads to the formation of highly reactive peroxynitrite (ONOO^−^), a potent driver of nitrative stress. This amplified oxidative and nitrative burden significantly contributes to exacerbation severity [36,37,38].

This pathway provides a plausible explanation of our findings. Tyrosine hydroxylase (TH), the rate-limiting enzyme in catecholamines synthesis, converts tyrosine to L-3,4-dihydroxyphenylalanine (L-DOPA) [39]. TH is highly vulnerable to both oxidative injury and inhibition by NO [40]. Given that COPD exacerbations are driven by increased inflammation and subsequent NO production, this process may cause tyrosine to accumulate due to TH inactivation. Furthermore, this NO-rich environment is clinically significant because peroxynitrite-mediated nitration inactivates histone deacetylase-2 (HDAC2), a key mechanism underlying corticosteroid resistance in COPD [38]. This proposed mechanism helps explain our observation that elevated tyrosine levels, reflecting a state of high nitrative stress and enzymatic blockade, were associated with an increased risk of COPD exacerbation.

It is also important to consider the complexity of tyrosine metabolism under stress. Under conditions of severe oxidative stress, hydroxyl radicals oxidize the benzyl ring of phenylalanine, leading to the production of abnormal tyrosine isomers, specifically meta-tyrosine and ortho-tyrosine. These isomers are recognized as biomarkers for hydroxyl radical attack and are themselves damaging to cells [41]. While our study utilized UPLC-based separation, its properties are comparable to high-performance liquid chromatography (HPLC), which cannot distinguish between co-eluting, structurally similar compounds like tyrosine, meta-tyrosine, and ortho-tyrosine [42]. Therefore, the detected signal likely serves as a ‘composite biomarker’ encompassing both native tyrosine and its oxidative isomers. While this limits chemical specificity, the potential contribution of these stress-induced isomers reinforces the biological link between this metabolic signal and the oxidative burden driving COPD exacerbations.

CRP levels serve as a general indicator of systemic inflammation across various conditions, including COPD [43]. Elevated CRP has been consistently observed in both stable and exacerbated COPD, where it has been linked to higher mortality risk and used to assess the response to antibiotic treatment during exacerbations [9,43,44,45]. Our finding that showed the CRP levels were linked to acute COPD exacerbations appears to contrast with the influential findings of Hurst et al., who found that past exacerbation history was a stronger predictor of future events than CRP levels [13]. However, our study resolves this apparent discrepancy by integrating CRP with a metabolic biomarker. The COPDAE score, created by combining CRP and tyrosine, demonstrated a significant association with future COPD exacerbations in multivariate analysis. Importantly, this prognostic value remained after accounting for GOLD group classification, which includes both exacerbation history and symptom severity. Therefore, the COPDAE score offers independent and additional prognostic stratification beyond established clinical indices, indicating that a multi-biomarker approach may improve risk assessment compared to single biomarkers.

To our knowledge, few studies have focused on the relationship between specific amino acid profiles and the prognosis of COPD exacerbations. In our analysis, we measured 19 amino acids using UPLC and identified tyrosine as the only amino acid with a potential association to exacerbation risk. This finding contrasts with those reported by Labaki et al., who identified lower concentrations of tryptophan, leucine, isoleucine, and valine as being independently associated with exacerbations within one year. However, several notable distinctions in the study design and population may explain these inconsistent results. In the Labaki study, only 58% of the participants met the formal criteria for COPD, whereas our cohort exclusively comprised COPD patients. Additionally, the mean age of participants in the Labaki study was significantly younger (53.7 years) compared to that in our study, which may indicate variations in disease stage or underlying metabolic conditions. Variations in diagnostic criteria and patient demographics likely explain differences in prognostic amino acids among COPD study populations.

In clinical practice, the COPDAE score could serve as a complementary biological profiler integrated into the current GOLD strategy. While the GOLD system relies heavily on retrospective history to guide initial treatment, the COPDAE score adds a prospective biological dimension. Specifically, for patients classified as ‘low risk’ (GOLD Group A or B) but presenting with a high COPDAE score (>1.73), clinicians might consider shortening follow-up intervals or initiating earlier interventions upon the onset of mild symptoms, rather than adhering strictly to the standard maintenance-only approach typically reserved for these groups.

This study has several limitations. First, the single-center study with modest sample size and a retrospectively determined cutoff value carries a risk of overfitting. Because internal validation was not feasible due to the limited number of events, the COPDAE score should be interpreted as exploratory and requires validation in larger, multi-center cohorts before clinical implementation. Furthermore, while the AUC of 0.668 indicates moderate discriminative ability, the score’s value lies in its potential to complement current clinical classifications by identifying ‘hidden’ risk in otherwise stable patients. Second, while tyrosine was identified as a prognostic factor, the underlying mechanism remains speculative, and the potential contributions of meta-tyrosine and ortho-tyrosine require further clarification. In addition, tyrosine was measured only once, and our UPLC method could not distinguish it from oxidative isomers, which limits biochemical specificity. Future studies using repeated measurements or high-resolution mass spectrometry are warranted. Third, due to the study design, limited sample size, and the homogeneous nature of our cohort, predominantly older male patients, we were unable to analyze the relationship between the COPDAE score and different COPD phenotypes or demographic subgroups. Future studies including more diverse patients should assess the score’s predictive value across specific exacerbation subtypes and phenotype-defined populations. Finally, the amino acid panel and other laboratory parameters were measured at one time point. This approach restricts the ability to rule out temporary fluctuations and does not fully capture the dynamic characteristics of the inflammatory process. Repeated longitudinal measurements are required to observe temporal variations in these biochemical mechanisms.

## 5. Conclusions

The COPDAE score, composed of tyrosine and HsCRP, is designed to assist in identifying and stratifying the risk of COPD exacerbations. This score may enable clinicians to improve risk assessment in stable COPD patients who present with fewer symptoms and a limited history of exacerbations, potentially recognizing individuals at increased risk who may not be detected by traditional assessment methods. This stratification could facilitate earlier reassessment and adjustment of medications to prevent potential future exacerbations. Although the COPDAE score appears useful for risk stratification, its impact on clinical outcomes following score-guided intervention remains unclear and will require further interventional studies. Additional research is also necessary to clarify the mechanistic role of tyrosine in COPD exacerbations.

## Figures and Tables

**Table 1:** Demographic and laboratory data in patients with chronic obstructive lung disease with or without composite event.

	All	Event (*n* = 44)	No Event (*n* = 44)	*p* Value
Age (years)	70.7 ± 9.8	71.5 ± 9.7	69.9 ± 9.9	0.434
Male (%)	78 (88.6)	36 (81.8)	42 (95.5)	0.089
Co-morbidity (%)				
Diabetes mellitus (%)	16 (36.4)	8 (18.2)	8 (18.2)	1.000
Hypertension (%)	47 (53.4)	26 (59.1)	21 (47.1)	0.285
Chronic kidney disease (%)	3 (3.4)	3 (6.8)	0 (0.0)	0.078
Atrial fibrillation (%)	7 (8.0)	4 (9.1)	3 (6.8)	1.000
Hyperlipidemia (%)	9 (10.2)	5 (11.4)	4 (9.1)	1.000
BMI (kg/m^2^)	23.9 ± 4.1	24.3 ± 4.6	23.4 ± 3.5	0.304
FEV1 (%)	53.1 ± 18.3	51.0 ± 18.6	55.1 ± 17.9	0.295
mMRC (Points)	1.9 ± 0.9	2.1 ± 0.9	1.6 ± 0.9	0.009
CAT (Points)	13.0 ± 8.3	14.3 ± 7.9	11.8 ± 8.7	0.149
6MWD (meters)	323.2 ± 127.0	303.3 ± 120.7	343.2 ± 131.3	0.142
Hand grip strength of dominant hand (kg)	29.8 ± 8.6	29.1 ± 9.3	30.5 ± 8.0	0.450
SpO2 (%)	96.5 ± 2.1	96.1 ± 2.3	97.0 ± 1.7	0.044
GOLD (%)				<0.001
A	18 (20.5)	4 (9.1)	14 (31.8)	
B	30 (34.1)	13 (29.5)	17 (38.6)	
C	15 (17.0)	6 (13.6)	9 (20.5)	
D	25 (28.4)	21 (47.7)	4 (9.1)	
BODE stages				0.241
1	33 (37.5)	12 (27.3)	21 (47.7)	
2	27 (30.7)	16 (36.4)	11 (25.0)	
3	17 (19.3)	9 (20.5)	8 (18.2)	
4	11 (12.5)	7 (15.9)	4 (9.1)	
COPD medication				0.056
LABA or LAMA	3 (3.4)	0	3 (6.8)	
LABA+LAMA	22 (25.0)	9 (20.5)	13 (29.5)	
ICS+LABA	14 (15.9)	5 (11.4)	9 (20.5)	
LABA+LAMA+ICS	49 (55.7)	30 (68.2)	19 (43.2)	
Additional oral steroid	8 (9.1)	7 (15.9)	1 (2.3)	0.058

Data are presented as mean ± standard deviation and number (%). BMI, body mass index; FEV1, forced expiratory volume in one second; mMRC, modified Medical Research Council dyspnea scale; CAT, chronic obstructive lung disease assessment test; 6MWD, six-minute walking distance; SpO2, oxygen saturation; GOLD, Global Initiative for Chronic Obstructive Lung Disease; COPD, chronic obstructive pulmonary disease; LABA, long-acting β adrenoceptor agonists; LAMA, long-acting muscarinic antagonists; ICS, inhaled corticosteroid.

**Table 2:** Comparisons and analysis of plasma amino acid concentrations and laboratory parameters between patients of chronic obstructive lung disease with and without composite events.

	Event (*n* = 44)	No Event (*n* = 44)	Univariate ^†^		Multivariate ^‡^	
			Hazard Ratio (95% CI)	*p* Value	Hazard Ratio (95% CI)	*p* Value
Amino acids (μM)						
Leucine	121.0 ± 24.1	121.8 ± 28.5	1.00 (0.99–1.01)	0.896		
Histidine	83.9 ± 19.3	85.0 ± 16.9	1.00 (0.98–1.02)	0.998		
Ornithine	96.1 ± 35.5	97.8 ± 26.6	1.00 (0.99–1.00)	0.899		
Phenylalanine	64.8 ± 17.2	61.6 ± 12.8	1.01 (0.99–1.03)	0.203		
Asparagine	54.6 ± 12.2	58.3 ± 14.4	0.99 (0.97–1.02)	0.508		
Taurine	48.4 ± 16.5	48.9 ± 18.6	1.01 (0.99–1.02)	0.510		
Serine	121.0 ± 28.8	125.7 ± 31.5	1.00 (0.99–1.01)	0.579		
Glutamine	667.1 ± 130.9	688.1 ± 140.1	1.00 (1.00–1.00)	0.602		
Arginine	34.4 ± 12.2	37.8 ± 13.0	1.00 (0.98–1.03)	0.790		
glycine	261.5 ± 79.2	242.6 ± 68.7	1.00 (1.00–1.00)	0.310		
Aspartic acid	3.1 ± 2.3	2.8 ± 2.6	1.04 (0.94–1.15)	0.410		
Threonine	138.8 ± 37.8	141.3 ± 45.1	1.00 (1.00–1.01)	0.582		
Alanine	424.6 ± 166.7	397.1 ± 133.1	1.00 (1.00–1.00)	0.152		
Proline	147.3 ± 57.6	160.2 ± 79.4	1.00 (1.00–1.00)	0.904		
Tyrosine	65.3 ± 15.4	61.9 ± 13.7	1.02 (1.00–1.04)	0.036	1.02 (1.00–1.05)	0.017
Methionine	32.8 ± 7.8	32.3 ± 7.2	1.03 (0.99–1.07)	0.109		
Valine	281.3 ± 61.4	273.6 ± 77.1	1.00 (1.00–1.01)	0.095		
Isoleucine	68.4 ± 17.7	69.7 ± 21.8	1.00 (0.99–1.02)	0.351		
Tryptophan	40.1 ± 10.6	41.9 ± 13.0	1.01 (0.98–1.03)	0.659		
Laboratory parameters						
HsCRP (mg/L)	2.6 (0.9–12.0)	1.3 (0.5–2.9) *	1.01 (1.00–1.04)	0.122		
Log (HsCRP)	0.5 ± 0.6	0.2 ± 0.5 *	1.54 (0.98–2.45)	0.064	1.66 (1.05–2.62)	0.029
Albumin (g/dL)	4.3 ± 0.4	4.4 ± 0.4	0.83 (0.45–1.52)	0.542		
Pre-albumin (g/dL)	28.3 ± 7.0	27.7 ± 6.7	1.02 (0.97–1.06)	0.474		
Transferrin (g/dL)	238.2 ± 39.1	242.2 ± 42.7	1.00 (1.00–1.01)	0.563		

Data are presented as mean ± standard deviation or median (interquartile range). * *p* < 0.05, compared to patients with composite events, by independent T test. ^†^ Univariate and multivariate analyses were performed by Cox regression. ^‡^ Multivariable analysis with forward selection model for all variables. CI, confidence interval; μM, micromolar; hsCRP, high-sensitivity C-reactive protein; Log, logarithm.

**Table 3:** Comparisons and analysis of COPDAE score and clinical parameters between patients with chronic obstructive lung disease, with and without composite events.

	Univariate ^†^		Multivariate ^‡^	
	Hazard Ratio (95% CI)	*p* Value	Hazard Ratio (95% CI)	*p* Value
Age	1.01 (0.98–1.04)	0.576		
Sex	0.54 (0.25–1.16)	0.114		
mMRC	1.79 (1.31–2.45)	<0.001		
GOLD	1.89 (1.41–2.54)	<0.001	1.94 (1.42–2.65)	<0.001
BODE	1.45 (1.08–1.94)	0.013		
SpO2	0.83 (0.73–0.95)	0.007		
COPDAE score	2.73 (1.43–5.21)	0.002	2.97 (1.39–5.95)	0.005

^†^ Univariate and multivariate analyses were performed by Cox regression. ^‡^ Multivariable analysis with forward selection model for all variables. CI, confidence interval; mMRC, modified Medical Research Council dyspnea scale; GOLD, Global Initiative for Chronic Obstructive Lung Disease; SpO2, oxygen saturation.

## Data Availability

The data that support the findings of this study are not publicly available due to restrictions imposed by Chang Gung Memorial Hospital. However, data are available from the corresponding author upon reasonable request and with the permission of Chang Gung Memorial Hospital.

## Abbreviations

The following abbreviations are used in this manuscript:

AUC	Area Under the Curve
BMI	body mass index
CAT	chronic obstructive lung disease assessment test
CIs	Confidence Intervals
COPD	Chronic Obstructive Pulmonary Disease
CRP	C-reactive protein
CV	coefficients of variation
EDTA	Ethylenediaminetetraacetic Acid
FEV1	forced expiratory volume in one second
6MWD	six-minute walking distance
GOLD	Global Initiative for Chronic Obstructive Lung Disease
HPLC	high-performance liquid chromatography
HRs	Hazard Ratios
hsCRP	high-sensitivity C-reactive protein
ICS	inhaled corticosteroid
LABA	long-acting β adrenoceptor agonists
LAMA	long-acting muscarinic antagonists
L-DOPA	L-3,4-dihydroxyphenylalanine
mMRC	modified Medical Research Council questionnaire
NO	nitric oxide
ROC	Receivers Operating Characteristic
ROS	Reactive Oxygen Species
SpO2	oxygen saturation
UPLC	ultra-performance liquid chromatography
